# iTRAQ-based quantitative analysis of age-specific variations in salivary proteome of caries-susceptible individuals

**DOI:** 10.1186/s12967-018-1669-2

**Published:** 2018-10-25

**Authors:** Kun Wang, Xiuqing Wang, Sainan Zheng, Yumei Niu, Wenyue Zheng, Xi Qin, Zhongcheng Li, Junyuan Luo, Wentao Jiang, Xuedong Zhou, Wei Li, Linglin Zhang

**Affiliations:** State Key Laboratory of Oral Diseases & National Clinical Research Center for Oral Diseases & Dept. of Cariology and Endodontics West China Hospital of Stomatology, Sichuan University, No. 14, Section 3 of Renmin South Road, Chengdu, Sichuan China

**Keywords:** Salivary proteome, Dental caries, Age, iTRAQ, MRM

## Abstract

**Background:**

Human saliva is a protein-rich, easily accessible source of potential biomarkers for the diagnosis of oral and systemic diseases. However, little is known about the changes in salivary proteome associated with aging of patients with dental caries. Here, we applied isobaric tags for relative and absolute quantitation (iTRAQ) in combination with multiple reaction monitoring mass spectrometry (MRM-MS) to characterize the salivary proteome profiles of subjects of different ages, presenting with and without caries, with the aim of identifying age-related biomarkers for dental caries.

**Methods:**

Unstimulated whole saliva samples were collected from 40 caries-free and caries-susceptible young adults and elderly individuals. Salivary proteins were extracted, reduced, alkylated, digested with trypsin and then analyzed using iTRAQ-coupled LC–MS/MS, followed by GO annotation, biological pathway analysis, hierarchical clustering analysis, and protein–protein interaction analysis. Candidate verification was then conducted using MRM-MS.

**Results:**

Among 658 salivary proteins identified using tandem mass spectrometry, 435 proteins exhibited altered expression patterns in different age groups with and without caries. Of these proteins, 96 displayed age-specific changes among caries-susceptible adults and elderly individuals, and were mainly associated with salivary secretion pathway, while 110 age-specific proteins were identified among healthy individuals. It was found that the age factor caused significant variations and played an important role in both healthy and cariogenic salivary proteomes. Subsequently, a total of 136 target proteins with complex protein–protein interactions, including 14 age-specific proteins associated with caries, were further successfully validated using MRM analysis. Moreover, non-age-specific proteins (histatin-1 and BPI fold-containing family B member 1) were verified to be important candidate biomarkers for common dental caries.

**Conclusions:**

Our proteomic analysis performed using the discovery-through-verification pipeline revealed distinct variations caused by age factor in both healthy and cariogenic salivary proteomes, highlighting the significance of age in the great potential of saliva for caries diagnosis and biomarker discovery.

**Electronic supplementary material:**

The online version of this article (10.1186/s12967-018-1669-2) contains supplementary material, which is available to authorized users.

## Background

Human saliva is mainly composed of the secretions from the parotid, submandibular, sublingual and minor salivary glands, with functional roles in protecting against tooth demineralization and microbial infection, participating in acquired enamel pellicle formation, and maintaining of the normal equilibrium buffer state [1–3]. Salivary proteins are a smaller component but are still crucial for reflecting the health status or disease susceptibility to oral and systemic pathologies. Alterations in salivary protein composition can be monitored using diagnostics techniques and compared with other clinical parameters. It is known that the composition of saliva varies with the human physiological states [4]. Changes in the production and concentration of saliva with aging have been reported [5]. Age-related variations in the salivary composition and gland morphology have been reported in healthy individuals [6]. Additionally, animal studies have revealed a reduction in salivary protein synthesis with aging [7]. A previous study compared the differences in the composition of the salivary proteome in healthy subjects stratified according to age by means of 2D-SDS-PAGE [8]. However, only limited and conflicting age-related differences have been noted. By contrast, another study reported no significant age-related changes in the salivary composition [9]. Therefore, a thorough understanding of whole saliva is a prerequisite for its diagnostic utility.

Dental caries is by far the most common multifactorial infectious oral disease, caused by complex interactions among cariogenic microorganisms, fermentable carbohydrates and many host factors, including saliva. As the host-associated factor, saliva plays an essential role in the dynamic equilibrium between demineralization and remineralization, and has been suggested to predict the development of caries [10]. Efforts to characterize whole saliva using two-dimension gel electrophoresis, HPLC combined with mass spectrometry, and immunoblotting have resulted in the identification of several salivary proteins associated with caries susceptibility [11, 12]. Vitorino et al. previously identified a strong correlation between salivary phosphopeptides and the absence of dental caries using HPLC–MS [13]. More recently, Castro et al. showed higher total protein concentration and salivary IgA level in caries-free adults compared to adults with high caries activity [14]. Nevertheless, as we know, the sensitivity and evolution of caries also depends on the influence of independent risk factors, including age, hygiene practices, education and income, which interact with the salivary components in a protective or a risk-increasing manner [15]. A previous study attempted to explore the relationship between several salivary components and dental caries, concluding that changes in salivary component output during aging are associated with a high caries prevalence [16]. However, limited studies identified only a few differentially expressed proteins without providing a comprehensive understanding of the effect of age on the relationship between changes in the salivary proteome and caries. To our knowledge, a comparative analysis of the human cariogenic salivary proteome along with aging has not yet been established.

With the rapid development of proteomics technology and application of high resolution MS, in-depth proteomic analyses have recently been achievable [17]. Isotope labeling techniques such as iTRAQ (isobaric tags for relative and absolute quantification) have been applied to the biomarker discovery and are particularly useful for quantitatively comparing proteomes between samples obtained from human. Moreover, the significantly shorter lead-time and reduced costs of the multiple reaction monitoring (MRM) assay make it a better alternative to immunoassays in protein biomarker validation to support preclinical and clinical studies [18, 19]. As a result of this proteomic technology, our group has already established a salivary proteome profile with a potential therapeutic use in preventing childhood dental caries [20]. Furthermore, to obtain an unrivalled understanding of the role of salivary proteins involved in caries resistance or cariogenicity, we performed a comparative analysis of salivary proteomes from caries-free to caries-susceptible subjects of different ages using iTRAQ coupled with MRM approach in this study.

In the present study, we aimed to (1) identify the salivary proteomic profiles of individuals of different ages presenting with and without dental caries; (2) characterize the changes in salivary proteome influenced by age and caries susceptibility; and (3) seek for age-specific and non-age-specific proteins associated with dental caries. For the first time, comparative salivary proteomics data were constructed for caries-susceptible young adults and the elderly, providing age-specific and non-age-specific candidates with diagnostic or protective value for caries susceptibility and caries prevention.

## Methods

### Subjects and saliva sampling

Written informed consent was obtained from all volunteers participating in this study, and the procedures were approved by the ethics committee of West China Hospital of Stomatology, Sichuan University (NO. WCHSIRB-D-2017-047), and conducted in accordance with the ethical guidelines. Saliva samples were randomly collected during follow-up clinical examinations at West China Hospital of Stomatology, according to the criteria defined by the World Health Organization [21], and then divided into four groups: 20 young adults aged between 19 and 24 years (mean age of 20.5 years) including caries-free (ACF, n = 10, DMFT = 0) and caries-susceptible (ACS, n = 10, DMFT ≥ 5) individuals, and 20 elderly subjects aged between 62 and 89 years (mean age of 82.8 years) including caries-free (ECF, n = 10, DMFT = 0) and caries-susceptible (ECS, n = 10, DMFT ≥ 5) individuals. A significant difference in the gender distribution was not observed among different groups. Inclusion criteria for all participating subjects with permanent dentition were an absence of severe systemic disorders and other detectable oral diseases, and no use of systemic antibiotics or antibacterial mouth rinse within the past 1 month.

Saliva collection was performed between 9:00 and 11:00 a.m. Subjects were informed in advance not to eat or to drink for at least 2 h before sampling [22]. A total of 3 ml spontaneous, whole unstimulated saliva was collected from each donor in a sterile enzyme-free conical tube according to the standard techniques [23]. After collection, all saliva samples were immediately transferred to the laboratory on ice. Supernatants were obtained through centrifugation at 12,000 rpm for 5 min at 4 °C, and then a protease inhibitor cocktail (Sigma-Aldrich, St Louis, MO, USA) was added to prevent proteolytic degradation within 2 h of collection [20]. After aliquoting, samples were stored at − 80 °C until further analyses.

### Extraction and trypsin digestion of whole salivary proteins

The salivary proteins from each group were pooled, reduced by adding 10 mM dithiothreitol and incubating at 56 °C for 60 min, and alkylated with 55 mM iodoacetamide at room temperature in the dark for 60 min. Afterwards, the treated proteins were precipitated in 80% acetone at − 20 °C for 3 h, and were then centrifuged at 20,000*g* for 30 min at 4 °C. The pellets were resuspended in 500 mM triethyl ammonium bicarbonate buffer containing 0.1% SDS. Each saliva pool was prepared using equal amounts of total protein from each group, and analyzed using SDS-PAGE. Duplicate aliquots of 100 µg of treated proteins were digested with trypsin (1:30 w/w, Promega, Madison, USA) overnight at 37 °C.

### iTRAQ labeling and SCX fractionation

For iTRAQ analysis, the tryptic peptides in each group of technical duplicates were labeled with iTRAQ reagents for 8-plex reaction according to the manufacturer’s instructions (Applied Biosystems, Foster City, USA), using 115 and 116 tags for the ACF group, 113 and 114 tags for the ACS group, 117 and 118 tags for the ECF group, and 119 and 121 tags for the ECS group, respectively. After 2 h of labeling reactions, each labeled peptide segments were mixed, further purified using Strata-X-C (Phenomenex, Torrance, USA), and then lyophilized with a Speed-vacuum to remove the reaction solvents. A detailed description of strong cation-exchange chromatography (SCX) fractionation is provided in Additional file 1.

### Identification of peptides using LC–MS/MS

Each SCX fraction was loaded twice onto a nano-RP column mounted on a Dionex ultimate 3000 nano-HPLC system (Shimadzu, Kyoto, Japan), and then eluted using an ACN gradient from 5 to 80% (v/v) containing 0.1% formic acid over 45 min at a flow rate of 300 nl/min. The eluates were directly injected into a Q-Exactive mass spectrometer (Thermo Fisher Scientific, Waltham, MA, USA), run in a positive ion mode with a full MS scan from 350 to 2000 m/z. The MS/MS spectra were acquired in a data-dependent mode and a high-sensitivity manner using the following parameters: full scans acquired at a resolution of 70,000 and MS/MS scans at a resolution of 17,500 with a minimum signal threshold of 1E+5.

### iTRAQ data processing and bioinformatics analyses

For iTRAQ-based protein identification, the original mass data were processed with Proteome Discover 1.3 (Thermo Fisher Scientific, Waltham, MA, USA; version 1.3) and searched with the Mascot search engine (Matrix Science, Boston, MA, USA; version 2.3.01) against the Uniprot protein database (http://www.uniprot.org) for *Homo sapiens* containing 20,199 sequences (release date 15.03.2016). Carbamidomethyl cysteine was specified as a fixed modification, with a tolerance of one missed cleavage site in the trypsin digests. Gln → pyro-Glu (N-term Q), oxidation (M), iTRAQ 8-plex (K), iTRAQ 8-plex (Y), and iTRAQ-8 plex (N-term) for methionine were selected as the potential variable modifications. The precursor mass tolerance was 15 ppm, and the product ion tolerance was 20 mmu. For protein identification, the following filters were used: significance threshold P < 0.05 (with 95% confidence) and ion score or expected cutoff less than 0.05 (with 95% confidence). For protein quantification, proteins for which at least one unique peptide was detected and the overall false discovery rate (FDR) was less than 1% were qualified for further analysis. The quantitative protein ratios were weighted and normalized to the median ratio in Mascot. Student’s t-test was performed to determine the significance of the differences in the levels of each protein when two groups were compared in each repetition. To be identified as being significantly differentially expressed, proteins were quantified in two biological replicates, along with a Fisher’s combined probability of < 0.05 and a fold change ± 1.2 (the average ratio of two repeat experiments). Additional filtering was performed using Bonferroni’s correction for multiple comparisons as necessary (P < 0.0125). The use of a conservative criterion was motivated by the goal of selecting a small subset of putative but reliable biomarkers.

Functional annotations of the identified proteins were conducted by performing gene ontology (GO) analysis of biological processes, molecular functions and cellular components. The Kyoto Encyclopedia of Genes and Genomes database (KEGG; http://www.genome.jp/kegg/pathway.html) was used to identify the majority of the important proteins involved in biological metabolic and signal transduction pathways. P values less than 0.05 were considered statistically significant using a two-tailed Fisher’s exact test. A hierarchical clustering (HCL) analysis of the quantitative data in the four comparative groups was performed using Cluster 3.0 software (Stanford University, USA) and visualized using Java Treeview software. The analysis of the protein–protein interaction (PPI) network was analyzed using the STRING online database (http://string-db.org) [24].

### Candidates verification using LC-MRM-MS

MRM assays were used to validate the differentially expressed proteins identified in the iTRAQ analysis. Details of the MRM analysis were referred to the procedure in our previous study [20] with some modifications as well as described in Additional file 1.

### Statistical analysis

Statistical calculations were performed using SPSS software version 19.0 (Chicago, IL, USA) and GraphPad Prism version 6.0 (San Diego, CA, USA). The unpaired Student’s t-test was employed to evaluate the differences between groups, which were considered statistically significant at P values less than 0.05.

## Results

### Overall workflow and iTRAQ-based quantification salivary proteomics

**Fig. 1:**
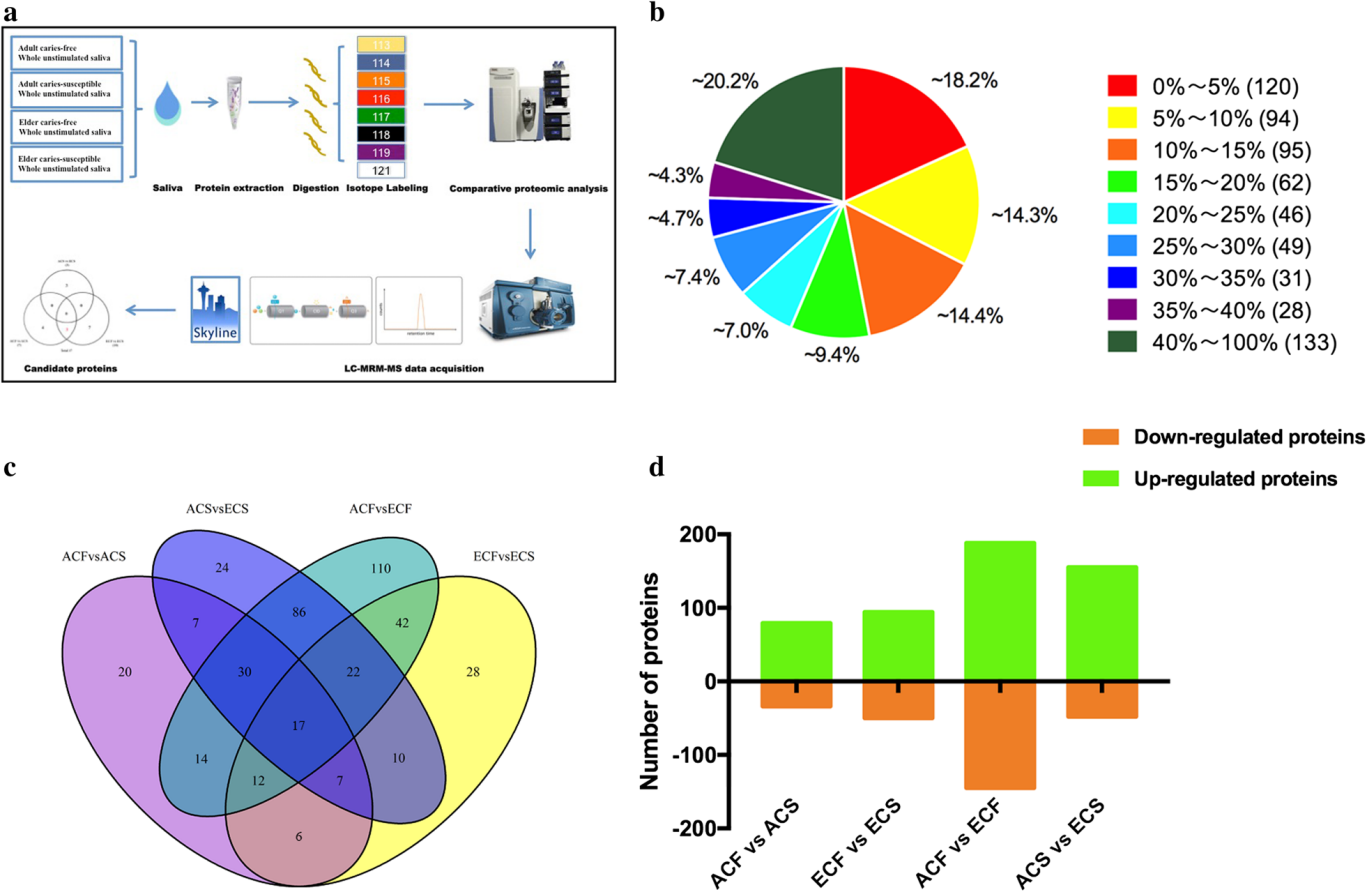
Experimental procedure and iTRAQ-based quantification of the salivary proteomes. **a** Workflow of the comparative analysis of salivary proteomes. **b** Distribution of the proteins’ sequence coverage. **c** Qualitative comparison of differentially expressed proteins identified commonly or exclusively among the four comparison groups. **d** The number of up- and down-regulated proteins in each comparison group

Four pooled saliva samples from the ACF, ACS, ECF, and ECS groups were used for this study. The demographic and clinical characteristics of all subjects are shown in Table 1 (Additional file 2: Table S1). The mean DMFT for two different aged groups showed no significant difference between males and females (P > 0.05). Quantitative proteomic analysis of whole saliva samples was performed using the iTRAQ-coupled LC–MS/MS method. The workflow of this study is illustrated in Fig. 1a. The protein composition of saliva samples from subjects of different ages presenting with and without caries showed visibly distinct band patterns via gel electrophoresis (Additional file 2: Figure S1), indicating the significance of the research regarding age-related changes in healthy and cariogenic human salivary proteomes.

**Table 1:** Demographics and caries state of all subjects

Group/gender	Number of subjects	Age (mean ± SD)	Male/female	DMFT (mean ± SD)
ACF	10	20.1 ± 0.57	5/5	0
ACS	10	20.9 ± 1.29	4/6	6.5 ± 1.90
ECF	10	80.9 ± 7.02	4/6	0
ECS	10	84.6 ± 4.38	5/5	9.1 ± 3.11
Adult male	9	20.78 ± 1.30	–	3.33 ± 4.27
Adult female	11	20.27 ± 0.79	–	3.18 ± 3.12
Elder male	9	84.67 ± 4.21	–	4.78 ± 4.79
Elder female	11	81.18 ± 6.94	–	4.36 ± 5.63
Total	40	51.63 ± 31.81	9/11	3.90 ± 4.42

Equal amounts of salivary proteins from four groups (100 μg for each group) with technical duplicates were pooled for the iTRAQ analysis. For protein quantification, the Pearson correlation analysis of the normalized tag intensities of spectra revealed a high reproducibility, with correlation coefficients exceeding 0.93 between technical duplicates from the four groups (Additional file 2: Figure S2). Thus, the spectra from technical duplicates were combined for further analysis. A total of 4564 unique peptides corresponding to 658 proteins (unique peptides ≥ 1) were identified, with a 1% false-positive rate at the protein level (Additional file 3). The distribution of the protein sequence coverage was analyzed for all proteins identified by iTRAQ (Fig. 1b). Based on these results, iTRAQ was able to cover the majority of the expressed salivary proteins. As for defining differentially expressed proteins, the criteria were established by P value < 0.05 and fold change > 1.2. Overall, 435 differentially expressed proteins were identified, 386 of which were significant after Bonferroni’s correction (P < 0.0125) (Additional file 4). For the quantitative analysis depicted in Fig. 1c, a Venn diagram was used to view the distribution of differentially expressed proteins and their overlaps among the four comparison groups. Of these, 17 proteins were commonly present in all comparisons, whereas 20, 28, 110, and 24 differentially expressed proteins were exclusively identified in the comparison groups of ACF vs ACS, ECF vs ECS, ACF vs ECF, and ACS vs ECS, respectively (Additional file 5). Importantly, the greatest number of differentially expressed proteins were identified in ACF vs ECF, followed by ACS vs ECS and ECF vs ECS, and the fewest number was identified in ACF vs ACS (Fig. 1d, Additional file 6). Compared with ECF group, 188 up-regulated proteins and 145 down-regulated proteins were identified in the ACF group, whereas 50 proteins displaying increased expression and 94 proteins exhibiting decreased expression were detected in the ECS group (Fig. 1d). Meanwhile, 79 up-regulated and 34 down-regulated proteins were identified in ACF compared to ACS group. Regarding the comparison between caries-susceptible young adults and elderly individuals, a total of 203 differentially expressed salivary proteins, including 155 overexpressed and 48 underexpressed proteins, were found in ACS group compared to ECS group (Fig. 1d).

### Comparative analysis of age-associated and caries-associated salivary proteins

**Fig. 2:**
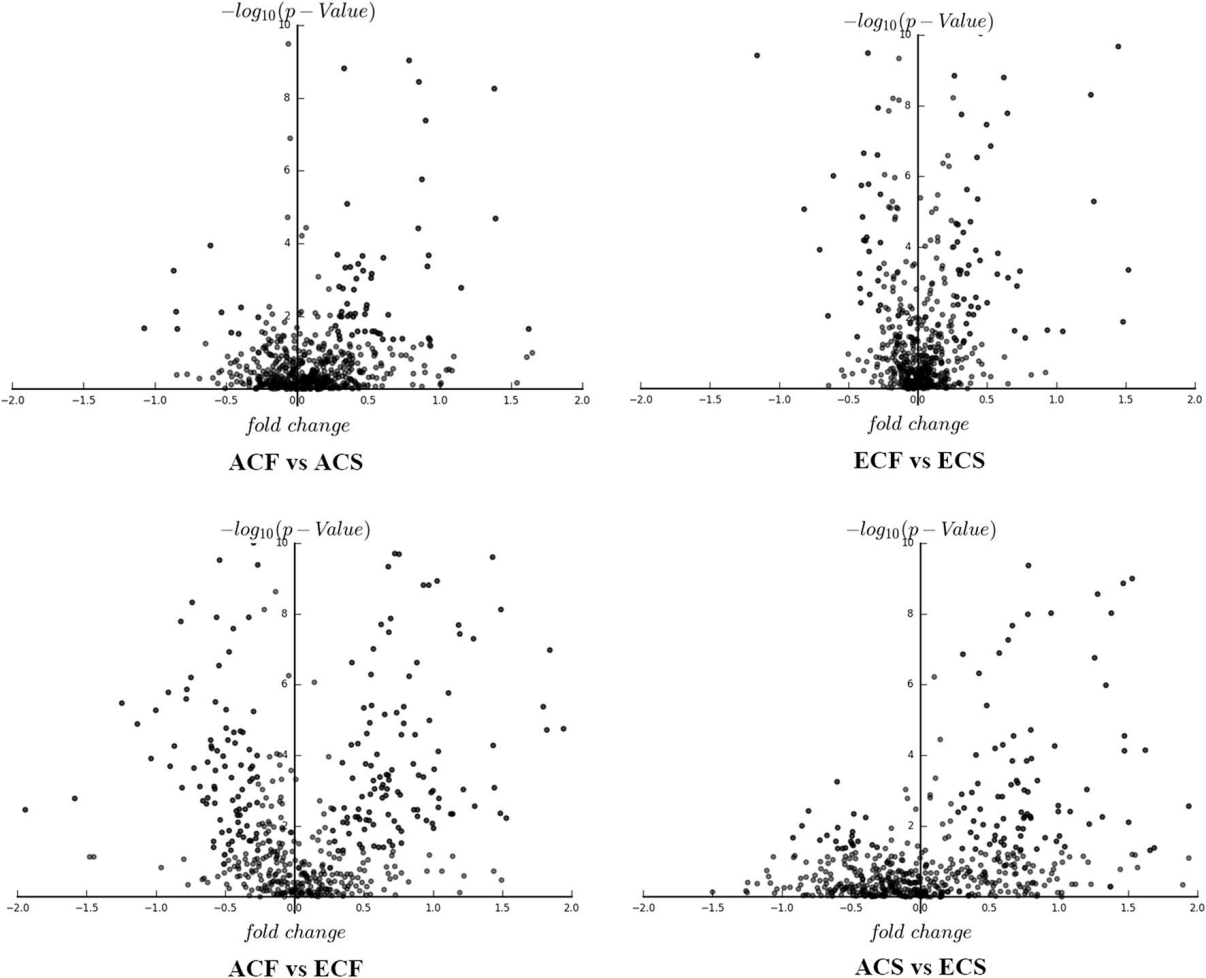
Volcano plots of differentially expressed proteins in comparison groups of ACF vs ACS, ECF vs ECS, ACF vs ECF, and ACS vs ECS

The influences of age and caries susceptibility on the human salivary proteome were then evaluated more comprehensively. According to the volcano plots of differentially expressed proteins, more significant variations in the salivary proteomes were observed between both age groups than between groups with and without caries (Fig. 2). Second, a far greater number of differentially expressed proteins was identified in both comparisons of different aged healthy groups and different aged caries-susceptible groups than that in the other two comparison groups. Furthermore, 110 specific differentially expressed proteins were identified between young adults and elderly individuals without caries, a number that was much more than the other three comparison groups (Fig. 1c). Therefore, age caused more significant alterations and played an important role in determining the caries-free and caries-susceptible human salivary proteomes.

### Comparative analysis of age-specific and non-age-specific salivary proteins associated with dental caries

**Fig. 3:**
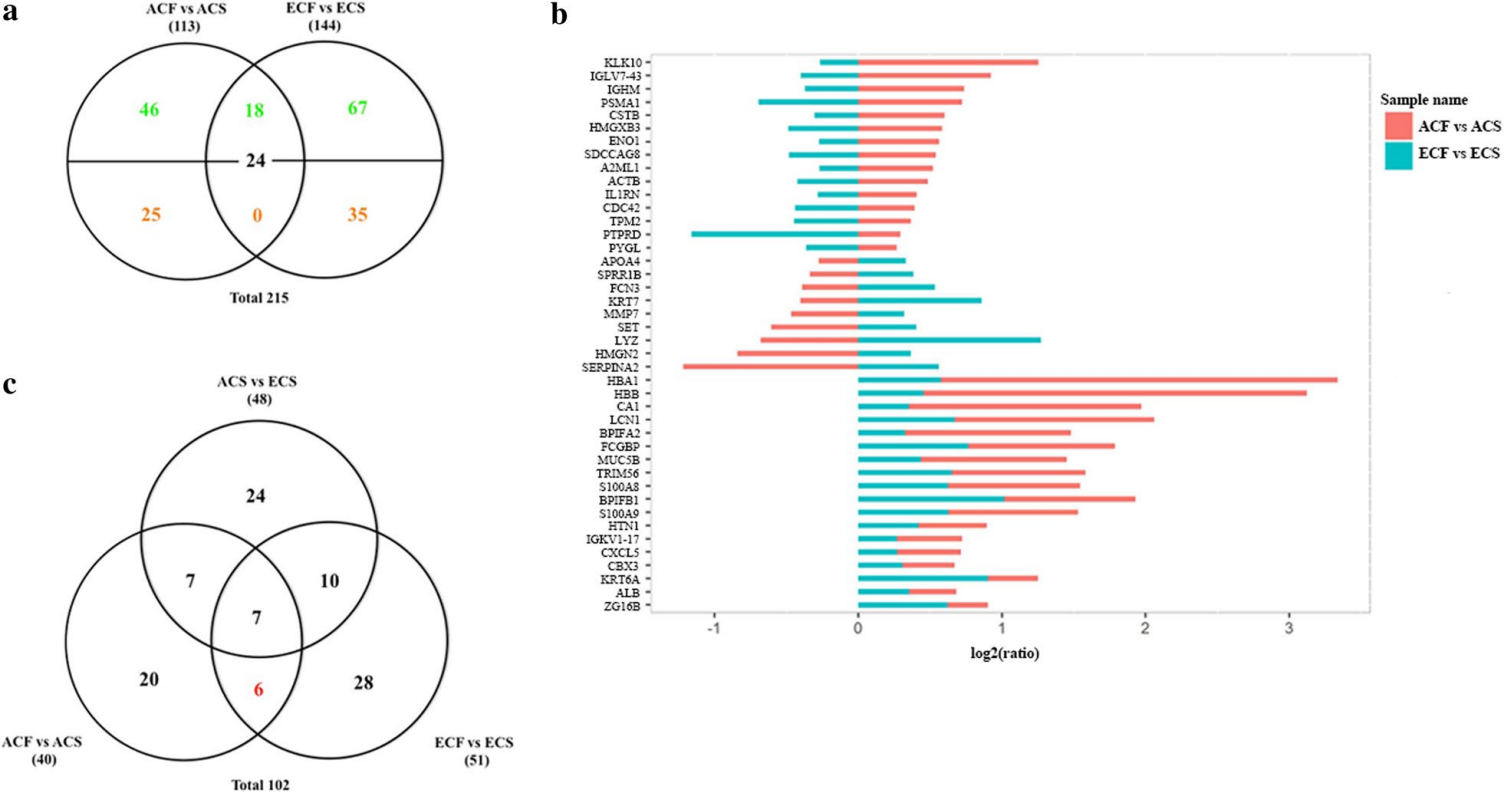
Comparison of differentially expressed proteins in ACF vs ACS, ECF vs ECS, and ACS vs ECS. **a** Evaluation of the overlap among the differentially expressed proteins derived from the iTRAQ analysis between ACF vs ACS and ECF vs ECS. **b** The relative fold changes (log2 (ratios)) of the tag intensity for differentially expressed proteins identified commonly in ACF vs ACS and ECF vs ECS. **c** Venn diagram showing differentially expressed proteins among ACF vs ACS, ECF vs ECS, and ACS vs ECS groups

Next, we conducted the comparison between ACF vs ACS and ECF vs ECS after mass spectrometry-based identification to further understand the age-specific alterations in salivary proteome of caries-susceptible individuals. Of the 215 differentially expressed proteins, 42 non-age-specific proteins overlapped between ACF vs ACS and ECF vs ECS. Among these proteins, 18 were detected in low levels in all patients of different ages with caries, such as histatin-1, BPI fold containing family B member 1, lipocalin-1, and protein S100-A9. The other 24 overlapping proteins showed different trends for changes in expression in the two comparison groups (Fig. 3a, Additional file 7). For example, cystatin B was detected at higher levels in saliva from ACF than ACS group, although it was present in lower amounts in ECF compared with ECS group. On the other hand, 71 specific differentially expressed proteins (46 up-regulated and 25 down-regulated) were identified in ACF vs ACS comparison group, and 102 unique differentially expressed proteins (67 up-regulated and 35 down-regulated) were identified in ECF vs ECS comparison group (Fig. 3a, Additional file 7). The log ratio of the relative intensity was illustrated to better visualize the salivary proteins that were differentially influenced by dental caries and aging (Fig. 3b, Additional file 2: Figure S3). Specifically, we also performed comparative analyses among ACF vs ACS, ECF vs ECS, and ACS vs ECS groups after excluding the proteins that overlapped with ACF vs ECF group. Eventually, 96 age-specific and 6 non-age-specific proteins were associated with dental caries, which were then selected for further validation (Fig. 3c, Additional file 8).

### Functional assessment of differentially expressed proteins among different comparison groups

**Fig. 4:**
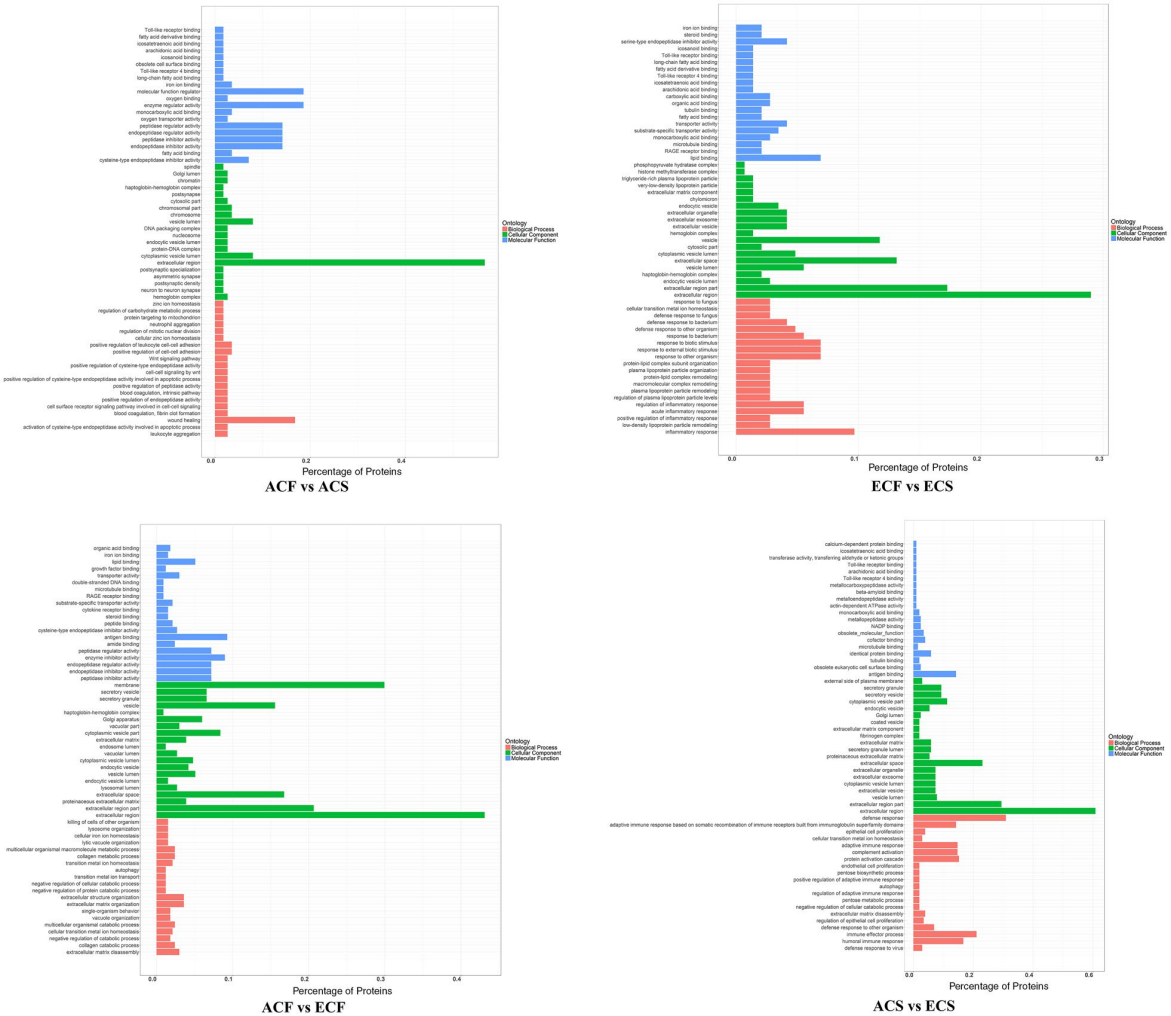
Gene ontology analysis of differentially expressed proteins in comparison groups of ACF vs ACS, ECF vs ECS, ACF vs ECF, and ACS vs ECS

**Fig. 5:**
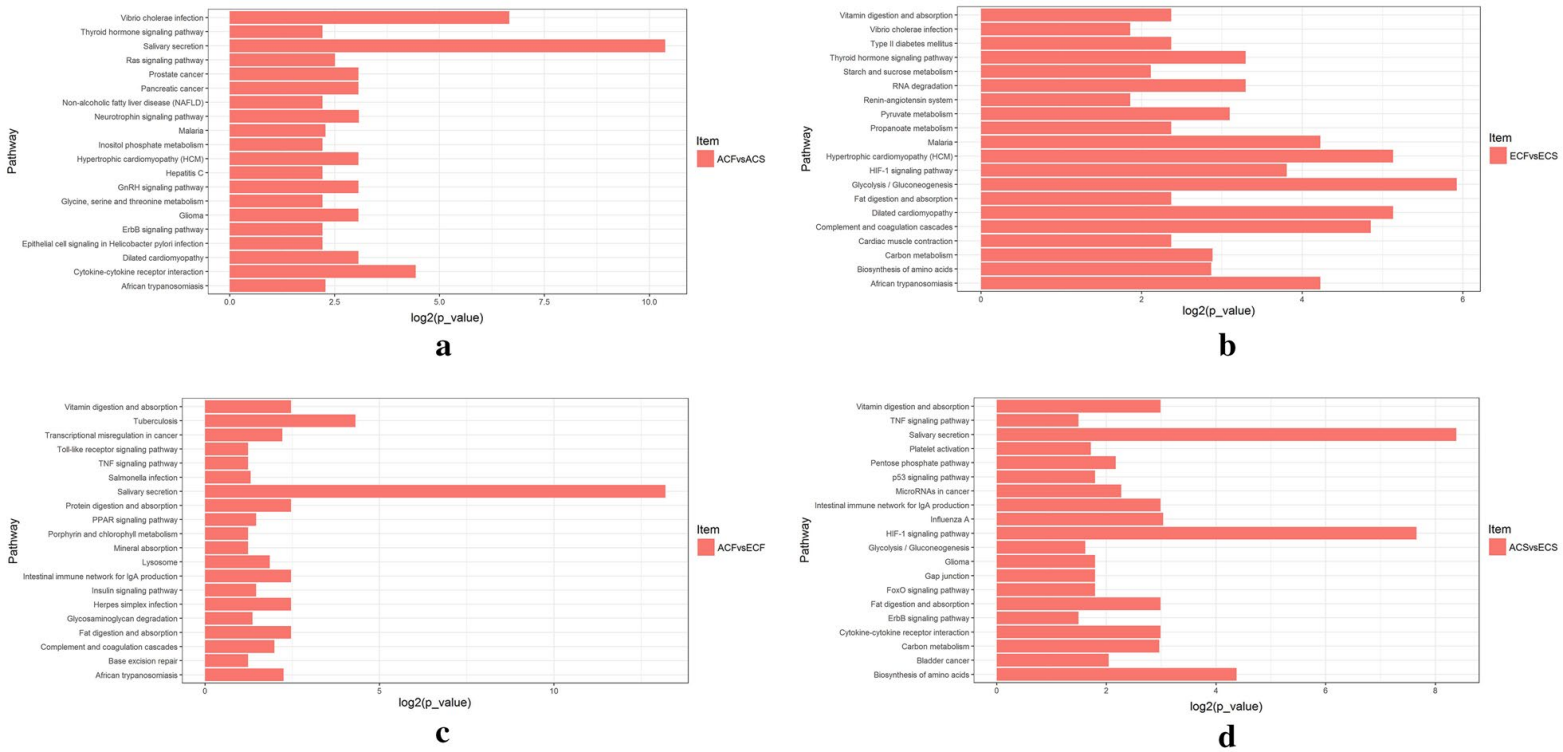
Pathway analysis of differentially expressed proteins in comparison groups of ACF vs ACS (**a**), ECF vs ECS (**b**), ACF vs ECF (**c**), and ACS vs ECS (**d**)

Gene ontology (GO) terms were further assigned to differentially expressed proteins according to the cellular components, molecular functions and biological processes (Fig. 4, Additional file 9). From the cellular component perspective, it can be noticed that the majority differentially expressed proteins in all comparison groups were located in the extracellular region (56.6% in ACF vs ACS, 29.2% in ECF vs ECS, 61.3% in ACS vs ECS, and 43.1% in ACF vs ECF). Regarding the molecular function, the differentially expressed proteins in both comparisons of different aged healthy groups and different aged caries-susceptible groups (ACF vs ECF, and ACS vs ECS) were involved in antigen binding (9.3%, 14.2%). Molecular function regulator (P value 0.036) and enzyme regulator activity (P value 0.026) were the top two significantly enriched terms in ACF vs ACS group, while lipid binding (P value 0.0004) and serine-type endopeptidase inhibitor activity (P value 0.014) were the top two significantly enriched terms in ECF vs ECS group. From the perspective of biological process, the most enriched terms were wound healing (P value 0.025) in ACF vs ACS, inflammatory response (P value 0.000059) in ECF vs ECS, extracellular matrix organization (P value 0.032) in ACF vs ECF, and defense response (P value 0.0057) in ACS vs ECS, respectively. Noticeably, in the KEGG pathway enrichment analysis, all the three comparison groups of ACF vs ACS, ACF vs ECF, and ACS vs ECS shared the same most significantly enriched pathway: salivary secretion. In addition, the top pathway in which differentially expressed proteins in ECF vs ECS group was involved was the gluconeogenesis pathway (Fig. 5, Additional file 10). Afterwards, a hierarchical clustering (HCL) analysis was used to reveal different features of age- and caries-associated salivary proteomes, nicely separating the experimental groups and their replicates into four comparison groups (Additional file 2: Figure S4). Moreover, the ACF vs ECF and ACS vs ECS groups were clustered together, while the ACF vs ACS and ECF vs ECS groups were grouped into a separate cluster. These results directly confirmed the visible difference between age and caries-associated salivary proteome profiling.

**Fig. 6:**
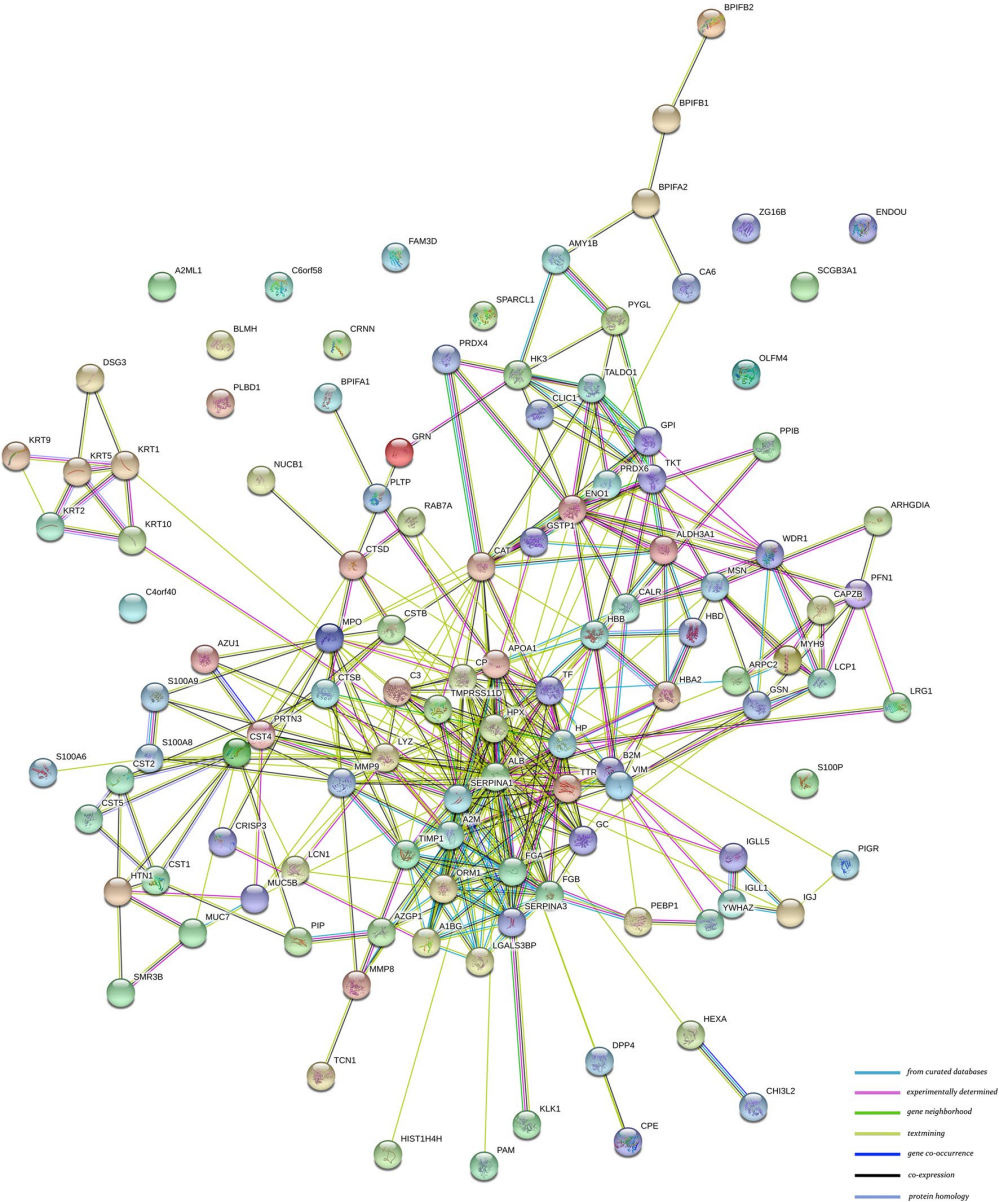
Interaction network of candidate proteins validated by MRM consisting of 115 proteins and 388 interactions

An integrated analysis of proteins expressed at different levels among the four comparison groups with successful validation was performed using the STRING online database, excluding proteins without information in the STRING database. The protein–protein interaction (PPI) network contained 115 proteins and 388 protein–protein interactions (Fig. 6, Additional file 11), in which alpha-1-antitrypsin and lysozyme C were key proteins and interacted with 24 and 19 proteins, respectively.

### Verification of candidate salivary proteins using MRM-MS

To further verify the reliability of the iTRAQ results, MRM analysis was performed. Consistent with the reported data [25, 26], as well as the differentially expressed proteins analyses, a total of 136 candidate proteins, including 36, 30, 56, and 113 proteins in comparison groups of ACF vs ACS, ECF vs ECS, ACS vs ECS, and ACF vs ECF, were selected and then successfully verified using an established MRM method (Additional file 12). Based on the results of MRM assay, the expression levels of these target proteins in different comparison groups were consistent with the iTRAQ expression patterns (Additional file 2: Figure S5, S6). The difference between the expression levels may have been due to the use of different detection methods.

Regarding the age-specific proteins in caries-free individuals, the MRM analysis validated 113 differentially expressed proteins in the ACF vs ECF group. Among them, 29 unique proteins, including 10 up-regulated and 19 down-regulated proteins, were further confirmed (Additional file 2: Figure S6). Compared with orally healthy elderly subjects, the MRM results revealed that the FAM3D and poly(U)-specific endoribonuclease proteins were up-regulated 3.40- and 2.67-fold, whereas apolipoprotein A-I and haptoglobin was down-regulated 12.76- and 9.01-fold in the saliva of young adults without caries, consistent with their expressed trends in the iTRAQ results (Additional file 2: Figure S6; Additional file 12). Additionally, the functional assessment showed that the up-regulated proteins in the ACF group were primarily involved in the immune response, complement activation, and serine-type endopeptidase activity, while the up-regulated proteins in the ECF group were mainly associated with neutrophil degranulation, metal binding, lipid metabolism, protein stabilization, and actin binding.

**Fig. 7:**
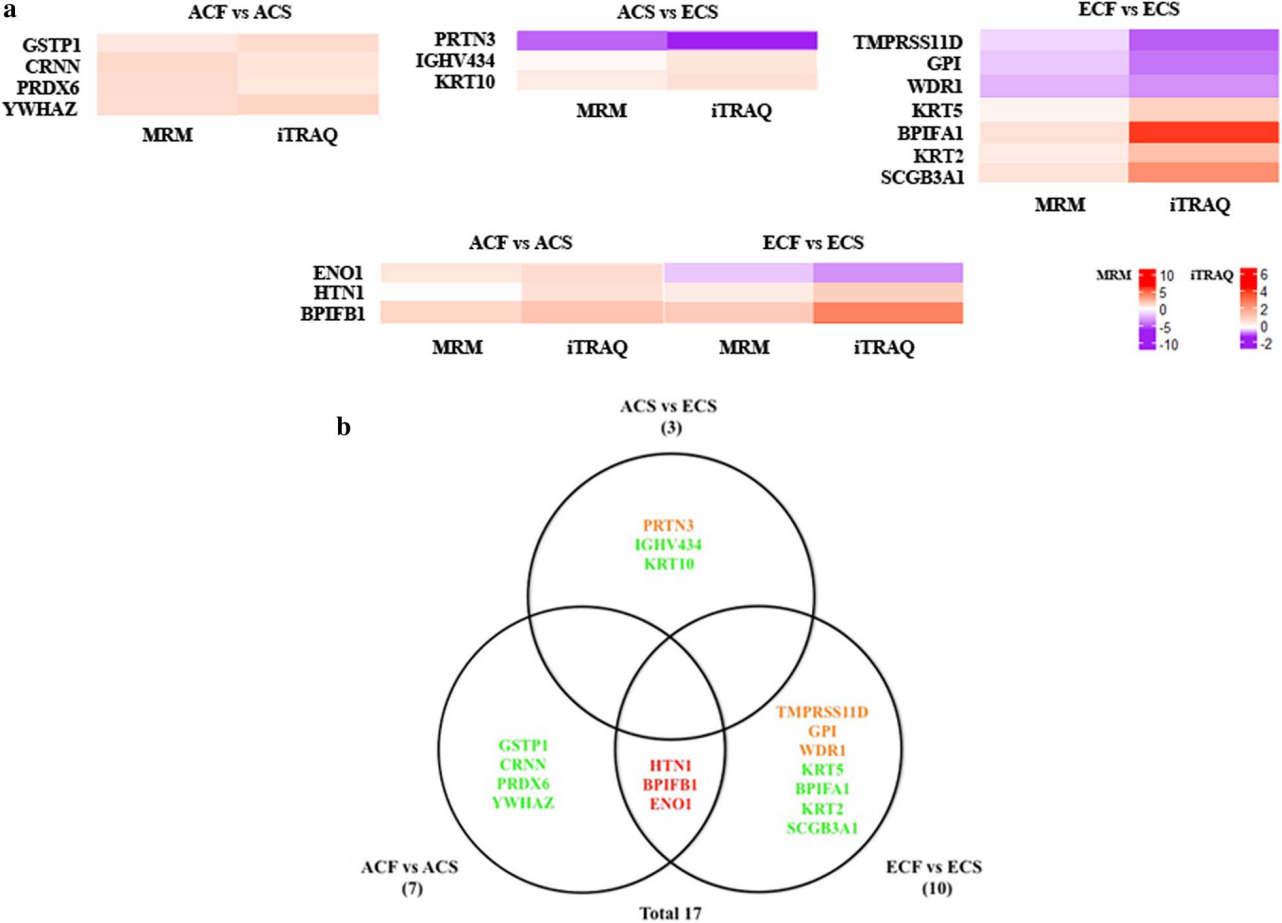
MRM verification of age-specific and non-age-specific proteins related to dental caries. **a** Heatmap illustrating the changes in the expression of age-specific and non-age-specific proteins in ACF vs ACS, ECF vs ECS, and ACS vs ECS groups measured by MRM and iTRAQ. **b** Venn diagram showing the differentially expressed proteins verified by MRM among ACF vs ACS, ECF vs ECS, and ACS vs ECS after excluding the proteins that overlapped with ACF vs ECF

For the analysis of age-specific and non-age-specific proteins in caries-susceptible individuals, MRM analysis verified 17 specific proteins among ACF vs ACS, ECF vs ECS, and ACS vs ECS groups after excluding proteins that overlapped with ACF vs ECF group. Fourteen of these age-specific proteins, such as cornulin, myeloblastin, keratin type II cytoskeletal 2 epidermal, keratin type II cytoskeletal 5, and keratin type I cytoskeletal 10, were confirmed to be associated with dental caries (Fig. 7, Additional file 13). The functional analysis showed that these age-specific proteins associated with dental caries were mainly involved in calcium ion binding, protein domain specific binding, cellular response to oxidative stress, keratinization, serine-type endopeptidase activity, antimicrobial humoral response, and regulation of immune response. On the other hand, 3 non-age-specific proteins, including histatin-1, BPI fold-containing family B member 1, and alpha-enolase were determined to be associated with dental caries (Fig. 7, Additional file 13). Importantly, histatin-1 and BPI fold-containing family B member 1 were down-regulated in both caries-susceptible young adults and elderly subjects compared to orally healthy controls, and were mainly associated with antimicrobial humoral response. By contrast, alpha-enolase showed different expression trends between the two comparison groups of ACF vs ACS and ECF vs ECS.

## Discussion

In this study, we analyzed the impact of age on the variations in caries-free and caries-susceptible human salivary proteomes based on a quantitative iTRAQ analysis. The proteomics data were constructed for four comparison groups of different ages and different caries-susceptibilities, and a total of 435 differentially expressed proteins were identified. These proteins exhibited a broad range of biological functions, with most involved in salivary secretion. In aspects of salivary gland function, flow rates of unstimulated whole saliva, as well as unstimulated and stimulated submandibular/sublingual saliva decreased with aging [27]. As we know, saliva serves multiple functions in the oral cavity, and its anti-caries activity depends on the remineralization, buffering, rinsing and anti-bacterial capacities. Decreased saliva secretion is considered a major problem related to caries prevalence [16]. Our results confirmed the consensus that human salivary secretion changes during aging and caries processes. In addition, we also compared the influences of age and caries susceptibility on the salivary proteome variations. Our finding implied that the effect of age on the salivary proteome was more significant than the caries status, highlighting an important effect of age on both healthy and cariogenic salivary proteomes.

During normal aging, the physiological states of the human body and oral microbial communities may change significantly, which might be conceivably cause variations in salivary proteome [28]. In the comparison between caries-free young adults and the elderly (ACF vs ECF), 110 unique proteins were identified from the iTRAQ results. Twenty-nine of these proteins were further verified using MRM analysis and were mainly associated with the immune response and complement activation. Our results were consistent with Sun et al. [29] who showed that salivary glycoproteins associated with aging were involved in the immune response and oral cavity protection. Therefore, these 29 age-related proteins might play a dominant role in the maintaining of oral health and homeostasis. In particular, apolipoprotein A-I showed the highest MRM relative abundance in the caries-free elderly individuals relative to young adults. Apolipoproteins have recently been suggested to be particularly relevant to the aging process and longevity by playing crucial roles in human immune functions [30].

When comparing the two groups between ACF vs ACS and ECF vs ECS, 18 common proteins with lower relative abundances in both caries-susceptible young adults and the elderly were identified, such as mucin-5B, histatin-1, BPI fold-containing family B member 1, protein S100-A9, protein S100-A8 and lipocalin-1, indicating their potential protective effects on dental caries. For example, mucin-5B has been implicated in the clearance of cariogenic bacteria in the oral cavity through reducing the attachment and biofilm formation of *Streptococcus mutans* [31]. Protein S100-A9 is a calcium- and zinc-binding protein with a prominent role in regulating the immune response and antimicrobial humoral response, and has also been reported to be associated with dental caries [32]. Protein–protein interactions are a common physiological mechanism for the protection and function of proteins in saliva. The STRING protein database was found to be useful for studying and predicting protein–protein associations [33]. In our previous study, the STRING online database, we identified 63 interactions in saliva samples from children with and without dental caries, and reported associations among histatin-1, mucin-5B, mucin-7, and cystatin S [20]. Consistent with these findings, the protein–protein interaction networks identified in the present study also corroborated this biological framework, highlighting their important roles in protecting the oral cavity across all ages. Therefore, the PPI network predicted from the STRING database in this study could provide a potentially useful platform for further exploration of the molecular mechanism underlying the complex interplay among different salivary proteins, which might be a more promising method for identifying caries-susceptible individuals. Notably, a separate network was predicted that contained the interactions of keratin type II cytoskeletal 1, keratin type II cytoskeletal 2 epidermal, keratin type II cytoskeletal 5, keratin type I cytoskeletal 9, keratin type I cytoskeletal 10, and desmoglein-3, which were mainly involved in the biological process of cornification and keratinization. Moreover, the down-regulation of keratin type II cytoskeletal 2 epidermal, keratin type II cytoskeletal 5, and keratin type I cytoskeletal 10 in caries-susceptible elderly individuals was also confirmed in the MRM analysis, potentially indicating an abnormal oral condition of those elderly subjects susceptible to dental caries. According to recent studies, a set of keratins was incorporated into mature enamel, and keratin 75 mutations are associated with increased susceptibility to dental caries [34, 35]. Keratins have the ability to spontaneously self-assemble and polymerize, facilitating the development of various types of biomaterials, such as porous scaffolds, films and hydrogels [35]. Combined with these supporting findings, our results indicated that keratins might potentially be used as novel tools for enamel repair and caries prevention, particularly for senile caries. However, additional studies are needed to validate these key screened proteins in a larger sample size, to further investigate the mechanism by which keratins protect against dental caries, and to translate these proteins from the laboratory level into clinical applications.

To further confirm the age-specific and non-age-specific salivary proteins related to dental caries, we narrowed the comparisons among ACF vs ACS, ECF vs ECS, and ACS vs ECS by excluding the differentially expressed proteins that overlapped with ACF vs ECF group. As a result of MRM, 14 age-specific proteins were verified to be associated with dental caries, among which glutathione *S*-transferase P, peroxiredoxin-6, WD repeat-containing protein 1, and glucose-6-phosphate isomerase exhibited complex interactions with each other. Their biological activities in the context of dental caries in subjects of different ages warrant future investigations. Additionally, as a serine protease involved in the antimicrobial humoral response, myeloblastin interacted with lysozyme C and mucin-5B, and was expressed at lower levels in caries-susceptible young adults than in the elderly. These results implied that the complex interaction among several salivary proteins and their functions associated with dental caries might differ in subjects of different ages in the way they act or in their degrees of importance, which should be considered distinct markers for adult and elderly population. In an attempt to put our results to good use, we propose that these age-specific proteins determined from an analysis of proteome variations in whole saliva could provide suitability for caries biomarker screening among different age groups. Of those non-age-specific proteins, histatin-1 and BPI fold-containing family B member 1, which are involved in the antimicrobial humoral response, were down-regulated in caries-susceptible young adults and the elderly compared to healthy controls. This result highlighted strong correlations between the absence of dental caries and high levels of histatin-1 and BPI fold-containing family B member 1. Histatin-1 is a major factor present in the protective proteinaceous structure on the tooth surface with antibacterial and antifungal activity, and displays a significant role in maintaining tooth integrity and protection against cariogenic bacteria [36, 37]. Interestingly, histatin-1 also showed decreased salivary abundance in caries-positive children in our previous study, and histatin-1 was suggested to be an important candidate biomarker of childhood caries [13, 20, 36]. To further provide stronger evidence for histatin-1 as a candidate biomarker applicable for detecting dental caries in all age groups, more in-depth studies on the intrinsic mechanisms underlying the functions of histatin-1 and the occurrence and development of dental caries will be performed in larger patient cohorts in the future.

## Conclusions

In summary, this study applied the discovery-through-verification pipeline to construct the first salivary proteomics map for individuals of different ages presenting with and without dental caries by means of iTRAQ/MRM technology. Our results indicated that age-specific differences existed in the unstimulated salivary proteome, and caused more significant variations in the salivary proteome than caries status. The obtained protein data from the present study contribute to improving our understanding of the effect of aging on the healthy and cariogenic salivary proteomes, highlighting the importance of age in the great potential of saliva for caries diagnosis and biomarker discovery. And the 14 age-specific proteins identified in this dataset hold not only important salivary candidates correlated with caries susceptibility in different age groups, but also serve as potential targets for preventive strategies against dental caries in the future. Moreover, two differentially expressed proteins, histatin-1 and BPI fold-containing family B member 1, were validated to be non-age-specific candidate biomarkers of dental caries, which may advance our utilization of salivary diagnostics for caries risk assessment.

## Additional files

**Additional file 1.** Detailed experimental protocols for the iTRAQ analysis and MRM validation.

**Additional file 2: Table S1.** Demographics and caries state of individual subject; **Figure S1.** SDS-PAGE gel electrophoresis of proteins from different saliva samples; **Figure S2.** Correlation analysis of the four experimental groups and their replicates in iTRAQ quantification; **Figure S3.** Comparison of the relative fold changes (log2 (ratios)) in the tag intensity of differentially expressed proteins identified exclusively in ACF vs ACS and ECF vs ECS groups; **Figure S4.** Hierarchical clustering analysis of differentially expressed proteins identified commonly in ACF vs ACS, ECF vs ECS, ACF vs ECF, and ACS vs ECS groups, as identified by iTRAQ-based LC–MS/MS. Saliva samples are shown in the columns, and proteins are demonstrated in the rows; **Figure S5.** Heatmap of the changes in the abundance of differentially expressed proteins in ACF vs ACS, ECF vs ECS, and ACS vs ECS groups measured using MRM and iTRAQ; **Figure S6.** Heatmap of the changes in the abundance of differentially expressed proteins and the proteins uniquely identified in the ACF vs ECF group, as measured using MRM and iTRAQ.

**Additional file 3.** List of proteins identified by iTRAQ in all saliva samples.

**Additional file 4.** List of significantly differentially expressed proteins with Bonferroni’s significance identified by iTRAQ in each comparison group.

**Additional file 5.** Category list of differentially expressed proteins commonly or uniquely detected in ACF vs ACS, ECF vs ECS, ACF vs ECF, and ACS vs ECS.

**Additional file 6.** List of differentially expressed proteins identified by iTRAQ in each comparison group.

**Additional file 7.** Category list of differentially expressed proteins commonly or uniquely detected in ACF vs ACS and ECF vs ECS.

**Additional file 8.** Category list of differentially expressed proteins commonly or uniquely detected in ACF vs ACS, ECF vs ECS, and ACS vs ECS after excluding proteins that overlapped with ACF vs ECF.

**Additional file 9.** Detailed information from GO analysis of differentially expressed proteins in ACF vs ACS, ECF vs ECS, ACF vs ECF, and ACS vs ECS.

**Additional file 10.** Category list of pathways involved by differentially expressed proteins in four comparison groups.

**Additional file 11.** Detailed information from the PPI analysis of target proteins.

**Additional file 12.** List of target proteins verified by MRM assay in each comparison group.

**Additional file 13.** Category list of verified proteins common or unique to the ACF vs ACS, ECF vs ECS, and ACS vs ECS after excluding proteins that overlapped with ACF vs ECF.
