# Supplementary material for: iTRAQ-based quantitative analysis of age-specific variations in salivary proteome of caries-susceptible individuals
Source: J Transl Med. 2018 Oct 25;16:293. doi: 10.1186/s12967-018-1669-2 (PMC6202833; doi:10.1186/s12967-018-1669-2)
Supplement: Supplementary file 1 — Additional file 1. Detailed experimental protocols for the iTRAQ analysis and MRM validation. [file 12967_2018_1669_MOESM1_ESM.docx]

**Experimental protocols**

**1. SDS-PAGE**

The salivary protein concentration was estimated using the Bradford protein assay (Bio-Rad, Hercules, California, USA). Whole saliva proteins were then analyzed using SDS-PAGE (sodium dodecyl sulfate-polyacrylamide gel electrophoresis). 10µl of each sample was mixed with loading buffer, boiled at 95°C for 8min, and applied onto a discontinuous 10% polyacrylamide gel. After SDS-PAGE, the bands on the gels were visualized via Coomassie brilliant blue staining.

2. **SCX fractionation**

The pooled peptides were dissolved in buffer A (10mM KH_2_PO_4_ in 25% ACN at pH 2.8). After adjusting the pH to 3 with H_3_PO_4_, the sample was fractionated using strong cation-exchange chromatography (SCX) on a HPLC (Shimadzu, Kyoto, Japan) equipped with a silica-based SCX column (250mm×4.6mm, Phenomenex, Torrance, CA, USA). The fractions were collected every 1min with a buffer B (10mM KH_2_PO_4_ and 2M KCl in 25% ACN, pH 2.8) gradient for 91min. A total of 16 fractions were desalted with a Strata-X 33µm PolyRevStage SPE (Phenomenex) following the manufacturer’s instructions and dried in Speed-vacuum, and then resuspended in 0.1% formic acid for analysis by LC-MS/MS.

**3. MRM validation of differentially expressed proteins from iTRAQ**

Approximately 2µg of digested peptides from saliva samples were analyzed on a TripleTOF 6600 System (AB SCIEX, Concord, USA) equipped with a nano LC system (Shimadzu, Kyoto, Japan) to identify target peptides with significant MS/MS signals corresponding to the protein candidates. The data were then searched against the Uniprot human sequence database, and the results were further imported to Skyline v2.1 (MacCoss Lab, University of Washington, Seattle, USA) to establish the MRM transition list. Ultimately, the selected transitions were adopted to survey the protein digests from saliva samples. All MRM samples from four groups were analyzed in triplicate using a QTRAP 6500 mass spectrometer (AB SCIEX, Framingham, USA) equipped with an ekspert nano LC 425 system (Eksigent, Silicon Valley, CA). The mobile phases consisted of solvent A (2% acetonitrile with 0.1% aqueous formic acid) and solvent B (98% acetonitrile with 0.1% formic acid). The peptides were separated on an eksigent column (75µm×15cm, 3µm particles, Eksigent, Silicon Valley, CA) at 300nL/min with a gradient of 5-30% solvent B for 45min, 30-80% solvent B for 4min, and maintenance at 80% for 5min. The MS parameters were set as follows: ion spray voltage at 2,400V, curtain gas at 35.0, ion source gas1 at 15.0, ion source gas2 at 0.0, collision gas at high, interface heater temperature at 150.0°C, entrance potential at 10.0, and Q1 and Q3 at unit resolution.

The obtained data was used skyline v2.1 software to integrate the raw file generated by QTRAP 6500 (AB SCIEX, Framingham, USA). To ensure the correct peak detection and integration, the data were manually inspected to further filter the peptides. The relative protein abundance of each target protein with two unique peptides was determined by averaging the two corresponding peptides.
