# Supplementary material for: iTRAQ-based quantitative analysis of age-specific variations in salivary proteome of caries-susceptible individuals
Source: J Transl Med. 2018 Oct 25;16:293. doi: 10.1186/s12967-018-1669-2 (PMC6202833; doi:10.1186/s12967-018-1669-2)

Additional file 2: Table S1. Demographics and caries state of individual subject.

Group	Sample No.	Age	Gender	DMFT
ACF	#1	20	F	0
ACF	#2	21	M	0
ACF	#3	20	F	0
ACF	#4	20	M	0
ACF	#5	19	F	0
ACF	#6	20	M	0
ACF	#7	20	F	0
ACF	#8	20	M	0
ACF	#9	21	M	0
ACF	#10	20	F	0
ACS	#11	20	F	6
ACS	#12	24	M	11
ACS	#13	21	F	7
ACS	#14	20	M	8
ACS	#15	20	M	5
ACS	#16	20	F	7
ACS	#17	21	F	5
ACS	#18	20	F	5
ACS	#19	22	F	5
ACS	#20	21	M	6
ECF	#21	62	F	0
ECF	#22	83	M	0
ECF	#23	83	F	0
ECF	#24	83	F	0
ECF	#25	79	M	0
ECF	#26	86	M	0
ECF	#27	81	F	0
ECF	#28	82	F	0
ECF	#29	83	F	0
ECF	#30	87	M	0
ECS	#31	78	F	14
ECS	#32	77	M	7
ECS	#33	86	F	13
ECS	#34	81	F	6
ECS	#35	86	M	11
ECS	#36	89	M	7
ECS	#37	88	F	10
ECS	#38	86	M	7
ECS	#39	89	M	11
ECS	#40	86	F	5

Additional file 2: Figure S1. SDS-PAGE gel electrophoresis of proteins from different saliva samples.

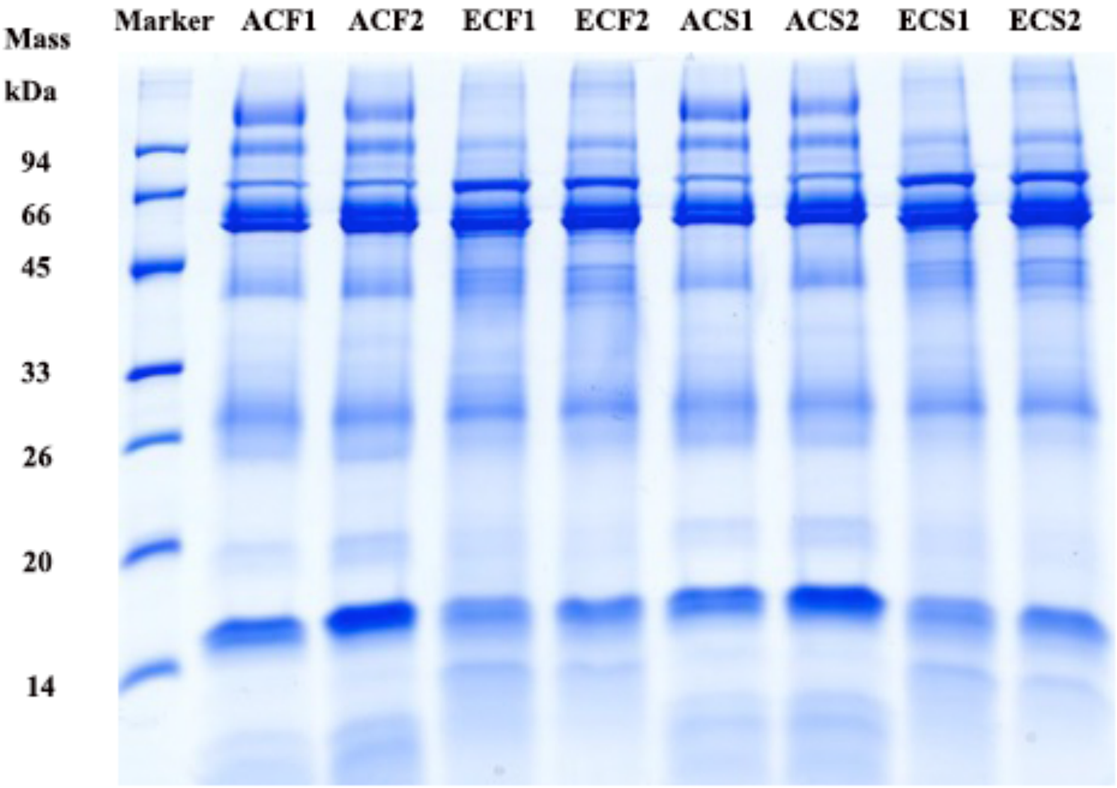

Additional file 2: Figure S2. Correlation analysis of four experimental groups and their replicate in iTRAQ quantification.

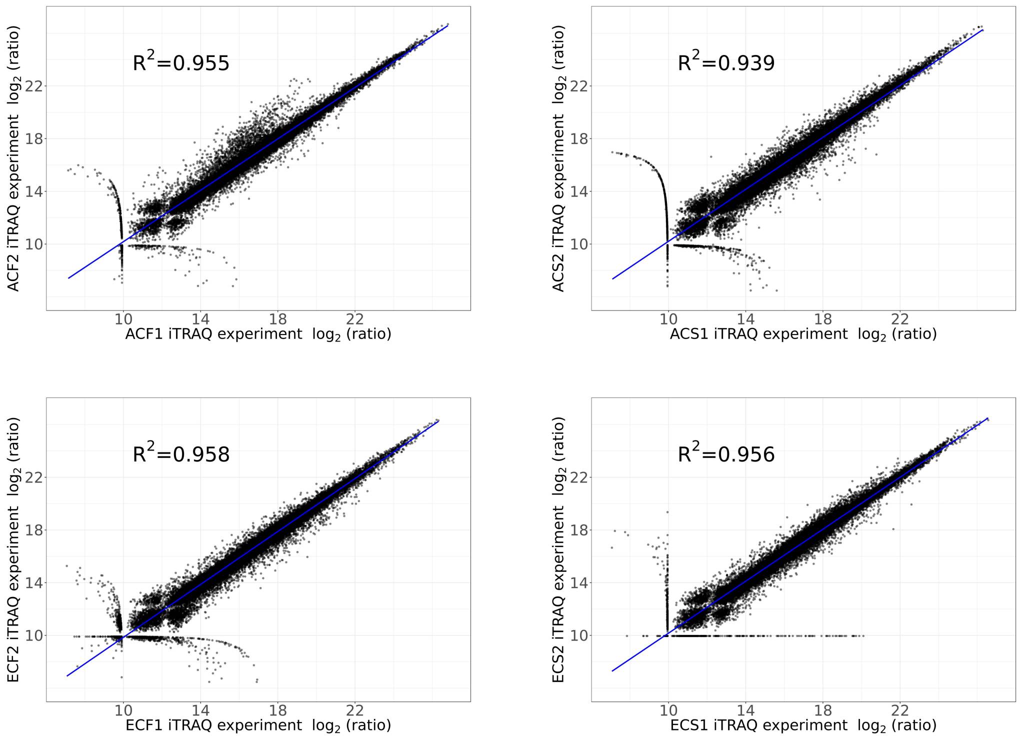

Additional file 2: Figure S3. Comparison of the relative fold changes (log2 (ratios)) of the tag intensity for differential proteins identified exclusively in ACF vs ACS and ECF vs ECS.

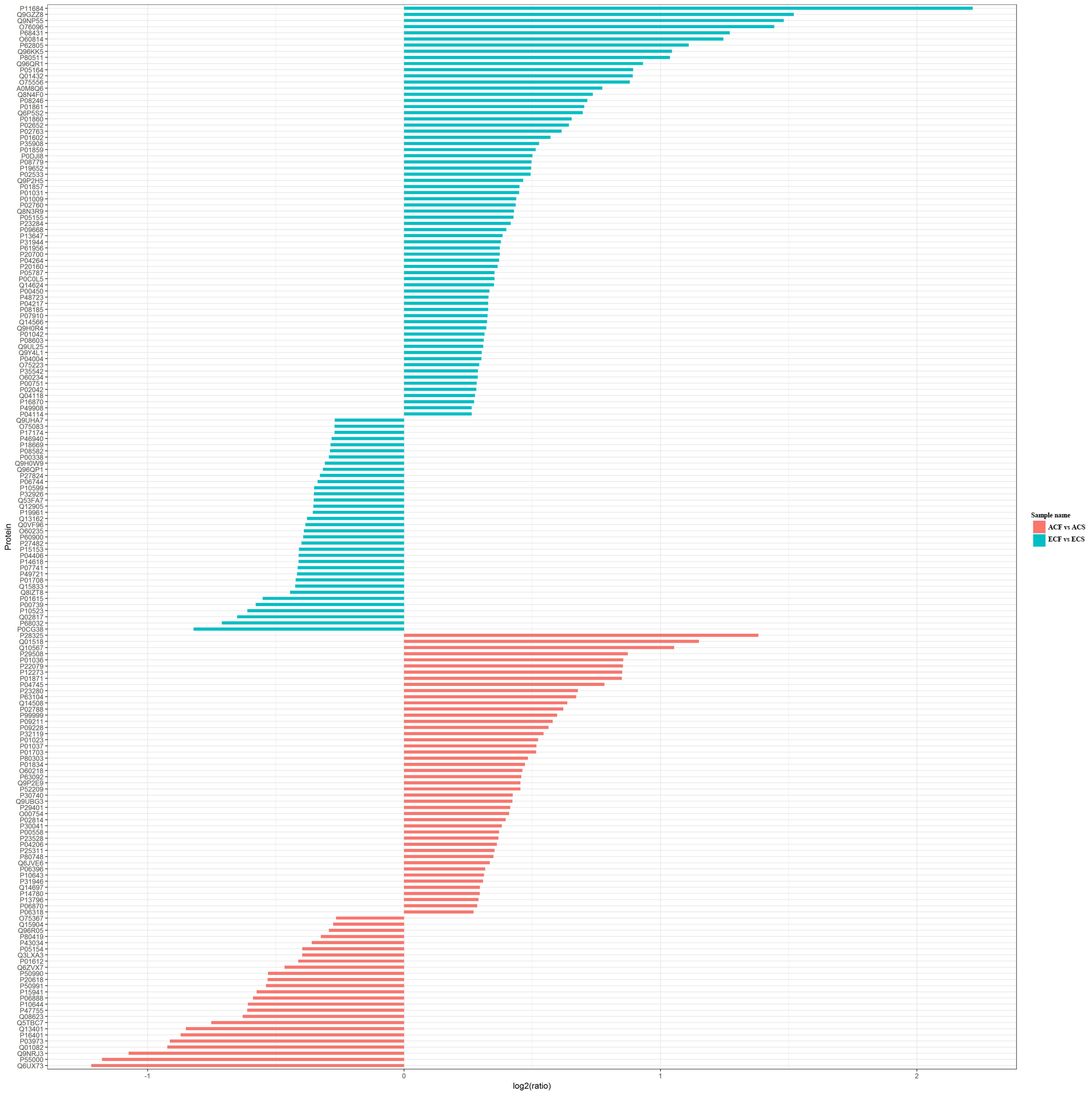

Additional file 2: Figure S4. Hierarchical clustering analysis of differentially expressed proteins identified commonly in ACF vs ACS, ECF vs ECS, ACF vs ECF, and ACS vs ECS by iTRAQ based LC-MS/MS. Saliva samples are shown in the columns, and proteins are demonstrated in the rows.

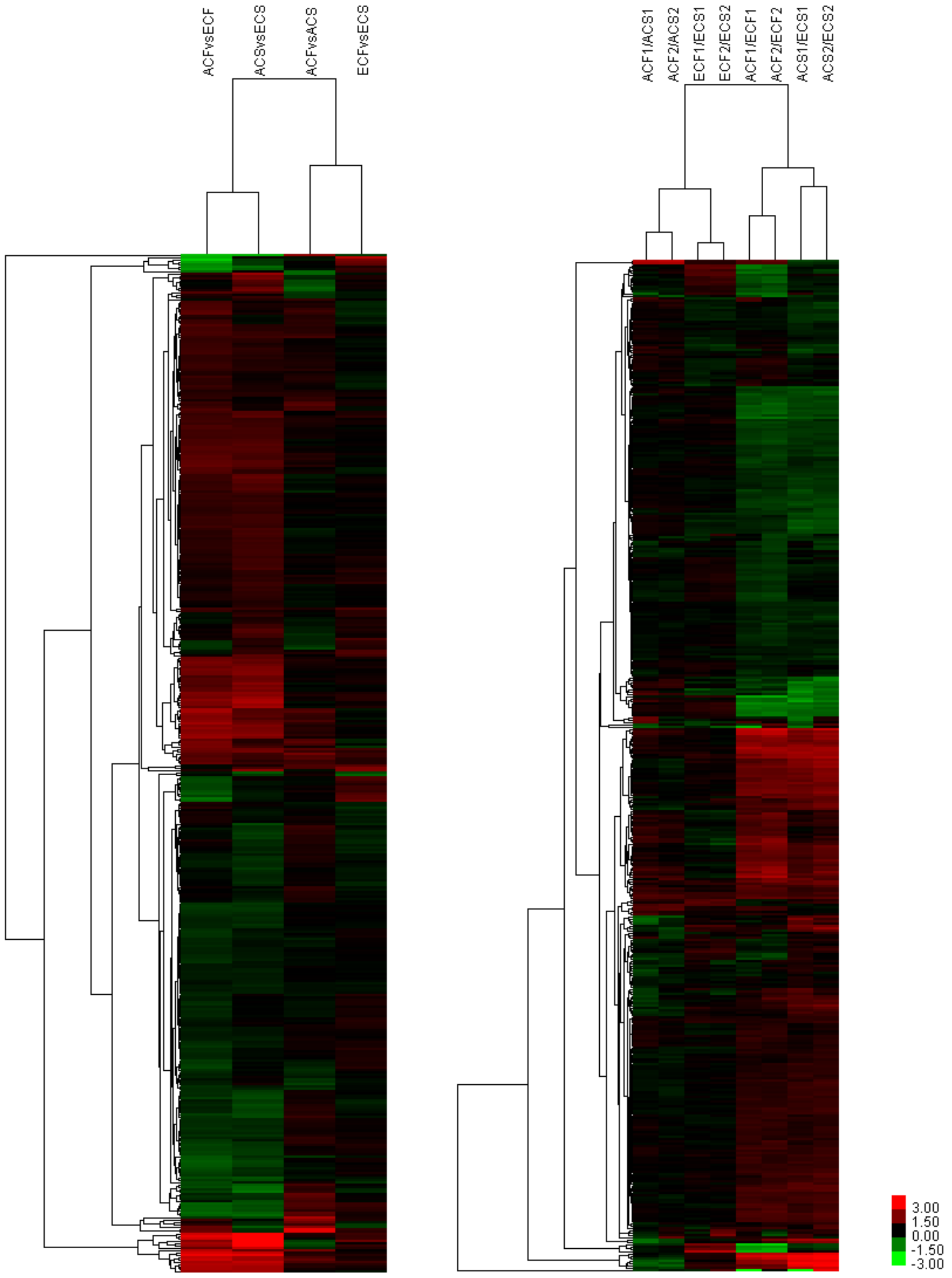

Additional file 2: Figure S5. Heatmap of the changes in abundance of differentially expressed proteins in ACF vs ACS, ECF vs ECS, and ACS vs ECS measured by MRM and iTRAQ.

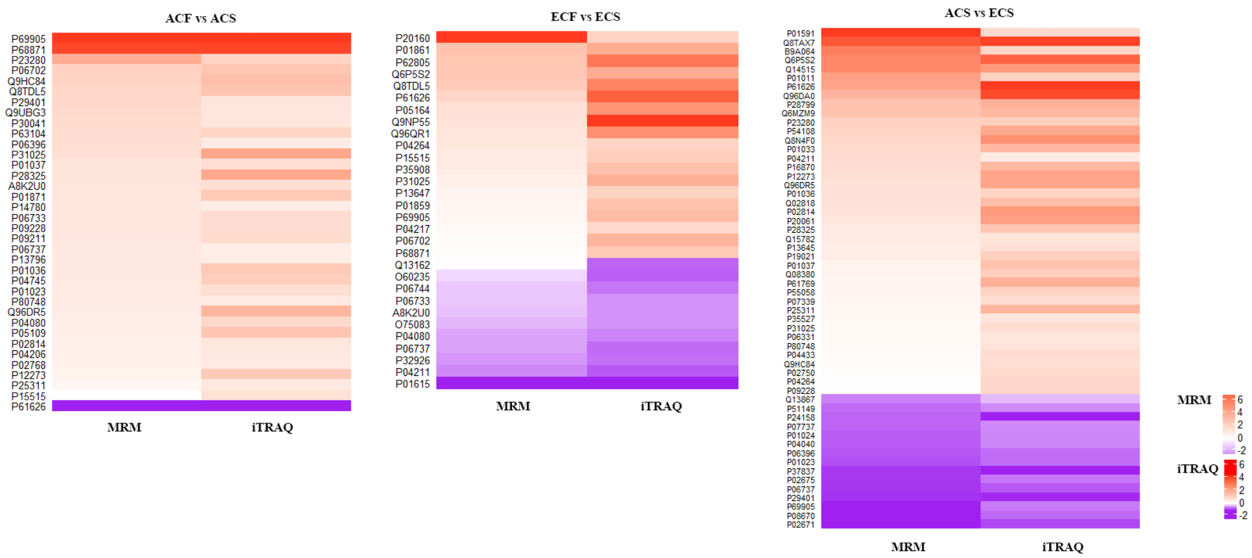

Additional file 2: Figure S6. Heatmap of the changes in abundance of differentially expressed proteins and the proteins uniquely found in ACF vs ECF, as measured using MRM and iTRAQ.

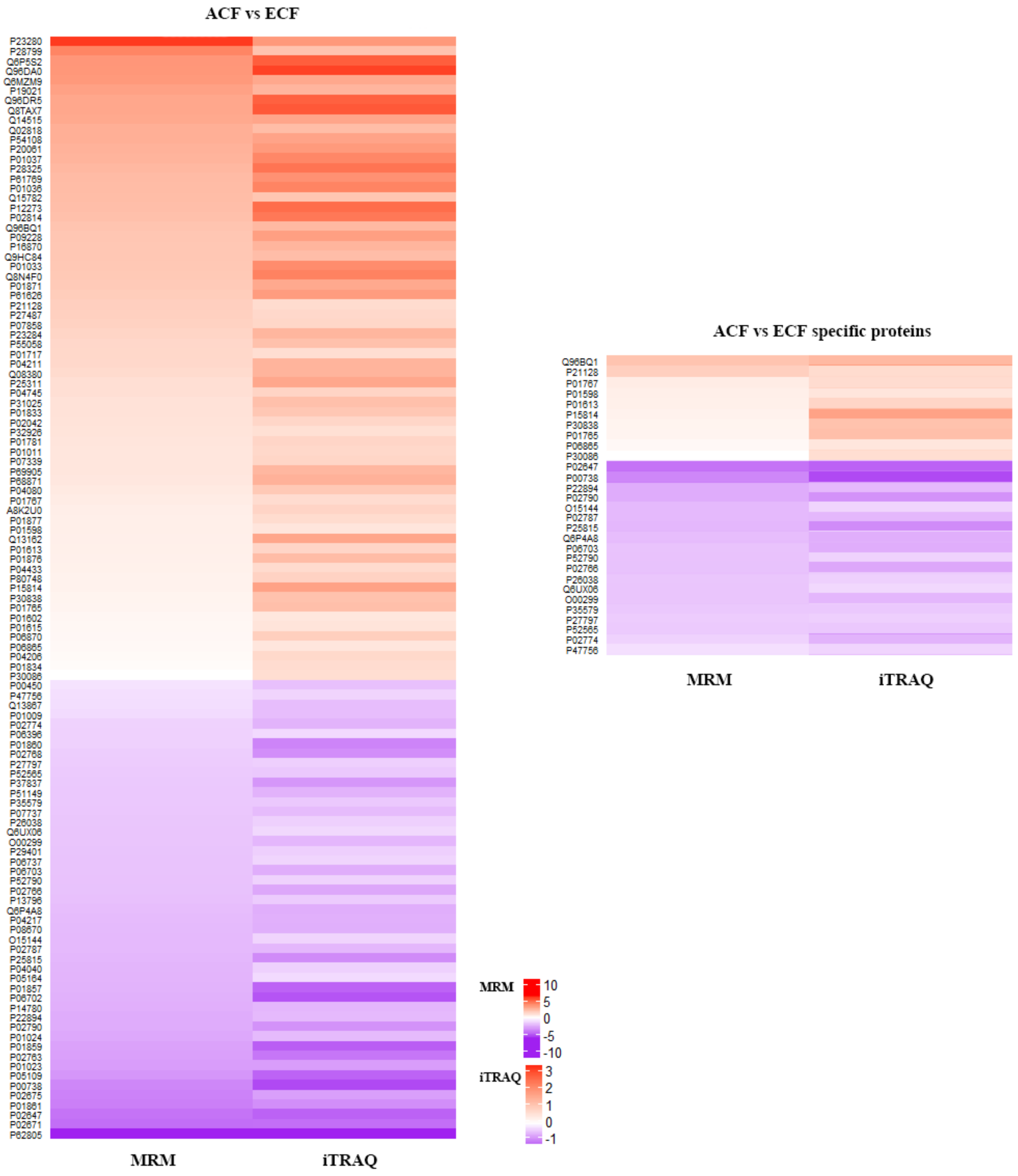

Supplement: Supplementary file 2 — Additional file 2: Table S1. Demographics and caries state of individual subject; Figure S1. SDS-PAGE gel electrophoresis of proteins from different saliva samples; Figure S2. Correlation analysis of the four experimental groups and their replicates in iTRAQ quantification; Figure S3. Comparison of the relative fold changes (log2 (ratios)) in the tag intensity of differentially expressed proteins identified exclusively in ACF vs ACS and ECF vs ECS groups; Figure S4. Hierarchical clustering analysis of differentially expressed proteins identified commonly in ACF vs ACS, ECF vs ECS, ACF vs ECF, and ACS vs ECS groups, as identified by iTRAQ-based LC–MS/MS. Saliva samples are shown in the columns, and proteins are demonstrated in the rows; Figure S5. Heatmap of the changes in the abundance of differentially expressed proteins in ACF vs ACS, ECF vs ECS, and ACS vs ECS groups measured using MRM and iTRAQ; Figure S6. Heatmap of the changes in the abundance of differentially expressed proteins and the proteins uniquely identified in the ACF vs ECF group, as measured using MRM and iTRAQ. [file 12967_2018_1669_MOESM2_ESM.pdf]
